# The Full Genome Sequences of Pseudomonas sp. Strain MM221 and *Pseudoarthrobacter* sp. Strain MM222, Isolated from a Meadow in Bielefeld, Germany

**DOI:** 10.1128/mra.01184-22

**Published:** 2023-01-04

**Authors:** Marion Eisenhut, Marian Hawrylo, Katerina Trevlopoulou, Hannah Fingerhut, Sophia Niemann, Lukas Brokate, Niklas Kniekamp, Christian F. Grenda, Marina Güttler, Lutz Wobbe, Andrea Bräutigam, Bianca Frommer

**Affiliations:** a Computational Biology, Faculty of Biology, Bielefeld University, Bielefeld, Germany; b Cluster of Excellence on Plant Sciences, Heinrich-Heine University, Düsseldorf, Germany; c Biology, Bielefeld University, Bielefeld, Germany; d Molecular Biotechnology, Bielefeld University, Bielefeld, Germany; e Center for Biotechnology, Bielefeld University, Bielefeld, Germany; University of Arizona

## Abstract

The bacterial strains Pseudomonas sp. strain MM221 and *Pseudoarthrobacter* sp. strain MM222 were isolated from a sandy soil sample. Here, we report on their complete genome sequences, including a circular plasmid for MM221, which were assembled after sequencing with an Oxford Nanopore Technologies flow cell.

## ANNOUNCEMENT

Microbial communities are major determinants in the soil environment. Their composition, diversity, and function are highly complex and poorly understood (1). To add genomic information on soilborne coinhabiting bacteria, we sequenced two independent bacterial isolates, MM221 and MM222, from a field sample.

We isolated both strains from a sandy soil sample that was collected in a meadow in Bielefeld, North Rhine-Westphalia, Germany (52°2′23.75016″N, 8°29′45.71592″E). The sample was taken from 5 cm below the surface of the ground, suspended in 0.9% (wt/vol) NaCl, filtered using a cellulose filter (pore size, 4 to 12 μm; product number 431015; Macherey-Nagel, Düren, Germany), and centrifuged. The cell pellet was resuspended in fresh 0.9% NaCl solution. Various dilutions were plated on agar medium (1.5% agar, 1% soy peptone, 0.3% NaCl, 0.1% sucrose, 0.1% cellulose, 0.1% xylan, 0.1% chitin, 0.05% Tris-HCl), and plates were incubated at 30°C for 7 days. Colonies of MM221 appeared yellow, and colonies of MM222 appeared white. Single colonies were picked, streaked on agar medium for propagation, and used for DNA isolation using the NucleoSpin microbial DNA kit (Macherey-Nagel) with RNA digestion according to the manufacturer’s manual. Barcoding of the genomic DNA was performed using a rapid barcoding kit (SQK-RBK004; Oxford Nanopore Technologies [ONT], Oxford, UK) according to the manufacturer’s protocol. For sequencing, one R9.4.1 flow cell was run for 15 h on a GridION system, and bases were called using the super-accurate base-calling model of MinKNOW v22.05.7 (all from ONT). The sequencing reads were processed for each barcode. All programs were run with default parameters unless otherwise specified. Adapters were trimmed with Porechop v0.2.3 (2), and BLAST was used to determine the expected genome sizes (3). The genome sequence was assembled as a single contig each with Canu v2.2 (4), using a genome size of 6.2 Mbp for Pseudomonas sp. strain MM221 and a genome size of 4.5 Mbp for *Pseudoarthrobacter* sp. strain MM222. For MM221, an additional contig for a plasmid was obtained. Assemblies were polished with Racon v1.5.0 (5), Minimap v2.24-r1122 with parameter setting –ax map-ont (6), and Medaka v1.6.0 with parameter setting –m r941_min_sup_g507 (ONT). Overlaps were trimmed with Berokka v0.2 (https://github.com/tseemann/berokka), and contigs were oriented according to *dnaA* with Circlator v1.5.5 (7). Genome completeness was examined with benchmarking universal single-copy orthologs (BUSCO) v5.4.3 with parameter setting –augustus (8). Genes were predicted with Prokka v1.14.5 (9). The organism and strain type were identified with TYGS (10). A metabolic pathway analysis was performed with KAAS (11), and potential antibiotic resistances were identified with the Comprehensive Antibiotic Resistance Database (CARD) (12). Relevant statistics for the raw reads and the genome sequences are listed in Table 1.

**TABLE 1:** Sequencing and assembly statistics for Pseudomonas sp. strain MM221 and *Pseudoarthrobacter* sp. strain MM222

Parameter	Finding for:	
	Pseudomonas sp. strain MM221	*Pseudoarthrobacter* sp. strain MM222
Raw sequencing reads		
No. of reads	74,793	48,962
Total length (bp)	487,682,423	311,527,256
*N*_50_ (bp)	11,033	11,142
Genome sequence		
Length (bp)	6,728,688	4,287,032
GC content (%)	61.95	66.46
Genome coverage (×)	72.48	72.67
Gene annotation		
Total no. of genes	6,534	3,934
No. of protein-coding genes	6,432	3,865
No. of rRNAs	22	15
No. of tRNAs	79	53
No. of transfer-messenger RNAs	1	1
BUSCO results (%)^*a*^		
Complete	93.3	99.8
Single copy	93.2	99.4
Duplicated	0.1	0.4
Fragmented	4.9	0.2
Missing	1.8	0.0
Plasmid		
Length (bp)	2,396	
No. of protein-coding genes	2	
GC content (%)	47.25	
Plasmid coverage (×)	329	

a The databases used (and the numbers of searched BUSCOs) were as follows: MM221, pseudomonadales_odb10 (782 BUSCOs); MM222, micrococcales_odb10 (537 BUSCOs).

**FIG 1:**
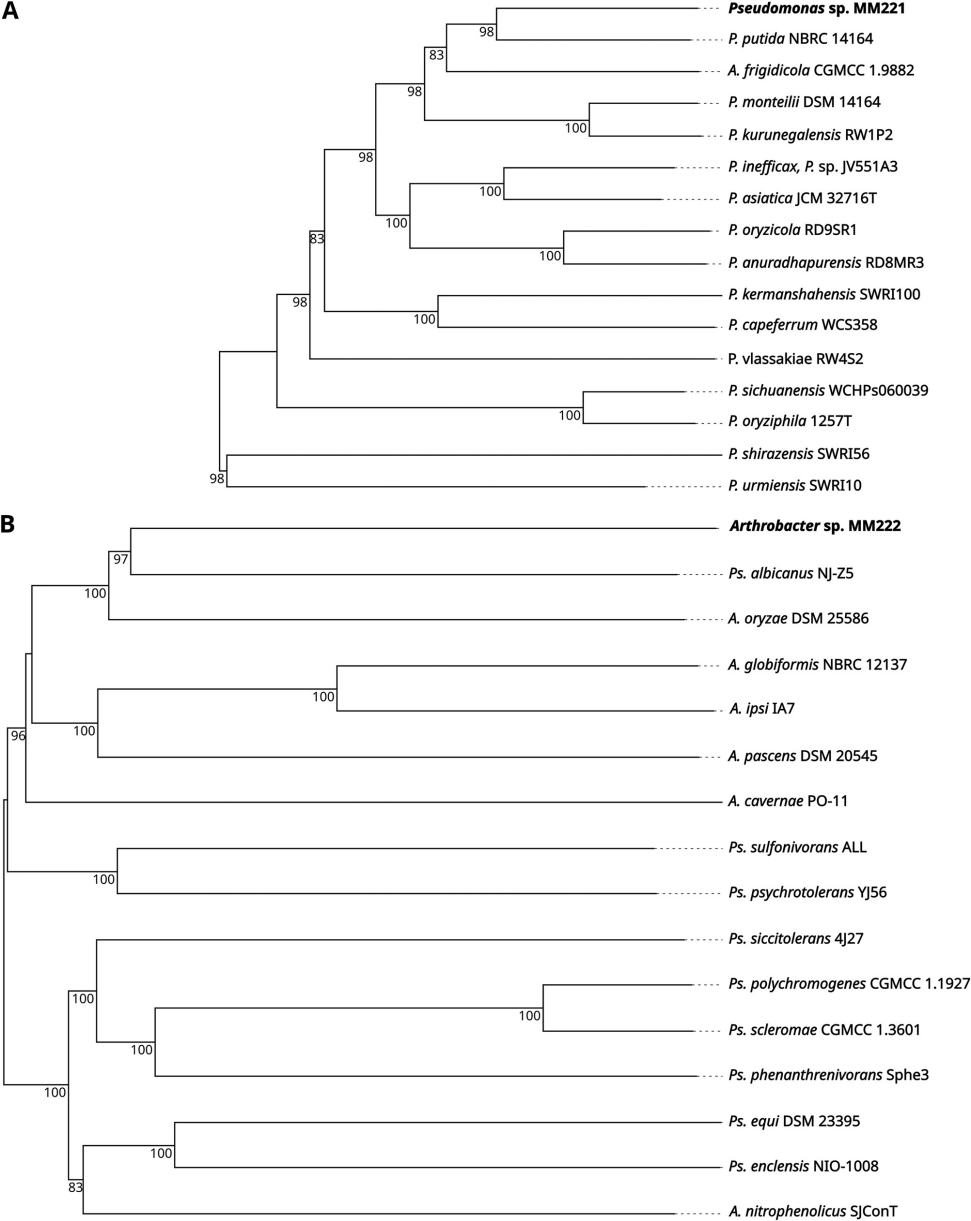
Genome BLAST distance phylogeny (GBDP) trees. Phylogenetic trees for Pseudomonas sp. strain MM221 (A) and *Arthrobacter* sp. strain MM222 (B) were constructed from genome sequences with TYGS (10). (A) The closest relative for MM221 is Pseudomonas putida NBRC 14164, with 51.2% digital DNA-DNA hybridization (dDDH) (formula d4) similarity (10). (B) With a dDDH (formula d4) value of 26.7%, Pseudoarthrobacter albicanus NJ-Z5 is the closest relative for *Pseudoarthrobacter* sp. strain MM222. The presented isolates MM221 and MM222 are highlighted in bold type. The pseudo-bootstrap support values from 100 replications are indicated at each branch point. *A*., *Arthrobacter*; *P*., Pseudomonas; *Ps*., *Pseudoarthrobacter*.

The genome of the presented Pseudomonas sp. strain MM221 has Pseudomonas putida NBRC 14164 (GenBank accession number NC_021505.1) (13) as the closest relative (Fig. 1A). A plasmid was coisolated and contains two open reading frames, encoding hypothetical proteins with unknown functions. According to CARD (12), the genome of Pseudomonas sp. strain MM221 contains genes conferring antibiotic resistance.

The other presented genome belongs to *Pseudoarthrobacter* sp. strain MM222, with Pseudoarthrobacter albicanus NJ-Z5 (GenBank accession number GCA_018224015.1) as the closest relative (Fig. 1B). A KAAS analysis (11) indicated that *Pseudoarthrobacter* sp. strain MM222 is likely auxotrophic for biotin.

### Data availability.

The MM221 and MM222 assemblies, gene annotations, and reads are available in GenBank/ENA under BioProject accession number PRJEB56332. The SRA accession number for the MM221 raw reads is ERS13525428, and the BioSample accession number for the MM221 assembly and annotation is ERS13579683. The SRA accession number for the MM222 raw reads is ERS13525429, and the BioSample accession number for the MM222 assembly and annotation is ERS13579684.

## References

[1] Fierer N. 2017. Embracing the unknown: disentangling the complexities of the soil microbiome. Nat Rev Microbiol 15:579–590. doi:10.1038/nrmicro.2017.87. 28824177

[2] Wick RR, Judd LM, Gorrie CL, Holt KE. 2017. Completing bacterial genome assemblies with multiplex MinION sequencing. Microb Genom 3:e000132. doi:10.1099/mgen.0.000132. 29177090 PMC5695209

[3] Johnson M, Zaretskaya I, Raytselis Y, Merezhuk Y, McGinnis S, Madden TL. 2008. NCBI BLAST: a better web interface. Nucleic Acids Res 36:W5–W9. doi:10.1093/nar/gkn201. 18440982 PMC2447716

[4] Koren S, Walenz BP, Berlin K, Miller JR, Bergman NH, Phillippy AM. 2017. Canu: scalable and accurate long-read assembly via adaptive *k*-mer weighting and repeat separation. Genome Res 27:722–736. doi:10.1101/gr.215087.116. 28298431 PMC5411767

[5] Vaser R, Sovic I, Nagarajan N, Šikic M. 2017. Fast and accurate de novo genome assembly from long uncorrected reads. Genome Res 27:737–746. doi:10.1101/gr.214270.116. 28100585 PMC5411768

[6] Li H. 2018. Minimap2: pairwise alignment for nucleotide sequences. Bioinformatics 34:3094–3100. doi:10.1093/bioinformatics/bty191. 29750242 PMC6137996

[7] Hunt M, Silva ND, Otto TD, Parkhill J, Keane JA, Harris SR. 2015. Circlator: automated circularization of genome assemblies using long sequencing reads. Genome Biol 16:294. doi:10.1186/s13059-015-0849-0. 26714481 PMC4699355

[8] Manni M, Berkeley MR, Seppey M, Simão FA, Zdobnov EM. 2021. BUSCO update: novel and streamlined workflows along with broader and deeper phylogenetic coverage for scoring of eukaryotic, prokaryotic, and viral genomes. Mol Biol Evol 38:4647–4654. doi:10.1093/molbev/msab199. 34320186 PMC8476166

[9] Seemann T. 2014. Prokka: rapid prokaryotic genome annotation. Bioinformatics 30:2068–2069. doi:10.1093/bioinformatics/btu153. 24642063

[10] Meier-Kolthoff JP, Göker M. 2019. TYGS is an automated high-throughput platform for state-of-the-art genome-based taxonomy. Nat Commun 10:2182. doi:10.1038/s41467-019-10210-3. 31097708 PMC6522516

[11] Moriya Y, Itoh M, Okuda S, Yoshizawa A, Kanehisa M. 2007. KAAS: an automatic genome annotation and pathway reconstruction server. Nucleic Acids Res 35:W182–W185. doi:10.1093/nar/gkm321. 17526522 PMC1933193

[12] Alcock BP, Raphenya AR, Lau TTY, Tsang KK, Bouchard M, Edalatmand A, Huynh W, Nguyen AV, Cheng AA, Liu S, Min SY, Miroshnichenko A, Tran HK, Werfalli RE, Nasir JA, Oloni M, Speicher DJ, Florescu A, Singh B, Faltyn M, Hernandez-Koutoucheva A, Sharma AN, Bordeleau E, Pawlowski AC, Zubyk HL, Dooley D, Griffiths E, Maguire F, Winsor GL, Beiko RG, Brinkman FSL, Hsiao WWL, Domselaar GV, McArthur AG. 2020. CARD 2020: antibiotic resistome surveillance with the Comprehensive Antibiotic Resistance Database. Nucleic Acids Res 48:D517–D525. 31665441 10.1093/nar/gkz935 PMC7145624

[13] Ohji S, Yamazoe A, Hosoyama A, Tsuchikane K, Ezaki T, Fujita N. 2014. The complete genome sequence of *Pseudomonas putida* NBRC 14164T confirms high intraspecies variation. Genome Announc 2:e00029-14. doi:10.1128/genomeA.00029-14. 24526630 PMC3924362

